# Swing Origami‐Structure‐Based Triboelectric Nanogenerator for Harvesting Blue Energy toward Marine Environmental Applications

**DOI:** 10.1002/advs.202401578

**Published:** 2024-04-11

**Authors:** Weilong Liu, Xiutong Wang, Lihui Yang, Youqiang Wang, Hui Xu, Yanan Sun, Youbo Nan, Congtao Sun, Hui Zhou, Yanliang Huang

**Affiliations:** ^1^ Key Laboratory of Advanced Marine Materials Institute of Oceanology Chinese Academy of Sciences Qingdao 266071 China; ^2^ Institute of Marine Corrosion Protection Guangxi Key Laboratory of Marine Environmental Science Guangxi Academy of Sciences Nanning 530007 China; ^3^ School of Mechanical and Automotive Engineering Qingdao University of Technology Qingdao 266525 China; ^4^ University of Chinese Academy of Sciences Beijing 100049 China

**Keywords:** cathodic protection, origami, swing structure, triboelectric nanogenerator, water wave energy

## Abstract

The appearance of triboelectric nanogenerators (TENG) provides a promising energy technology for harvesting abundant water wave energy. Here, the design and fabrication of a swinging origami‐structured TENG (SO‐TENG) tailored specifically for water wave energy harvesting are presented. The design incorporates an oscillating structure weighted at the bottom, inducing reciprocating motion propelled by the inertia of passing water waves. This reciprocating motion efficiently converts mechanical into electrical energy through the origami structure. By employing origami as the monomer structure, the surface contact area between friction layers is enhanced, thereby optimizing output performance. the swinging structure, combined with the placement of heavy objects, enhances the folding and contact of the origami, allowing it to operate effectively in low‐frequency water wave environments. This configuration exhibits robust power generation capabilities, making it suitable for powering small electronic devices in water wave environments. Furthermore, when applied to metal corrosion protection, the SO‐TENG demonstrates notable efficacy. Compared to exposed Q235 carbon steel, Q235 carbon steel protected by SO‐TENG exhibits a significant reduction in open‐circuit potential drop, approximately 155 mV, indicative of superior anti‐corrosion properties. It lays a solid foundation for water wave energy collection and self‐powered metal corrosion protection in marine environments.

## Graphical abstract

A triboelectric nanogenerator (TENG) based on a swing origami structure is successfully developed, which has excellent electrical output performance and device stability. Swinging origami (SO)‐TENG devices have good electrical output performance. It can be applied to self‐powered service systems. It shows that TENG has broad application prospects in large‐scale wave energy harvesting and self‐powered service systems in the marine environment.

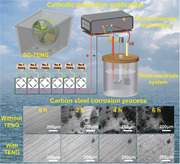

## 1 Introduction

With the continuous development and utilization of fossil energy, the degree of energy crisis has gradually intensified, and the ecological environment has been deteriorating.^[^ ^1^, ^2^ ^]^ Consequently, the development and utilization of renewable clean energy have become paramount. marine energy, abundant on earth, holds immense potential for obtaining marine environmental information and monitoring the marine environment.^[^ ^3^ ^]^ To this end, researchers have invested in the research and development of energy harvesting in marine environments. Presently, energy harvesting devices in marine environments primarily rely on traditional electromagnetic generators (EMGs).^[^ ^4^ ^]^ However, their low energy conversion efficiency, high cost, and large footprint hinder their application in the marine environment.^[^ ^5^, ^6^ ^]^ Therefore, it is necessary to develop cost‐effective marine energy harvesting devices with high energy conversion efficiency.

Recently, the research of triboelectric nanogenerators (TENG) has provided a new strategy for various kinds of energy harvesting.^[^ ^7^, ^8^, ^9^, ^10^, ^11^, ^12^, ^13^ ^]^ Compared with traditional EMG, TENGs have obvious advantages, such as the simplicity of their design and their small footprint.^[^ ^14^, ^15^, ^16^, ^17^, ^18^ ^]^ Various energy harvesting types of TENG have been proposed to capture energy from human activity,^[^ ^19^, ^20^ ^]^ wind,^[^ ^21^, ^22^, ^23^, ^24^, ^25^ ^]^ water waves,^[^ ^26^, ^27^, ^28^, ^29^, ^30^, ^31^ ^]^ and raindrops.^[^ ^32^, ^33^ ^]^ At present, various TENG structures have been designed specifically for water wave energy harvesting. In the marine environment, water wave movement is random and unpredictable. Driven by this motion, TENGs with different configurations exhibit various movements, including swinging,^[^ ^34^ ^]^ slapping movement,^[^ ^35^, ^36^ ^]^ sliding movement,^[^ ^37^ ^]^ and rolling ball‐type independent layer movement.^[^ ^38^, ^39^ ^]^ In addition, the surface charge density and surface contact area between friction layers can affect the output performance of TENG.^[^ ^40^ ^]^ To increase the surface charge density, many researchers have explored extensively and modified the friction layer material to increase the electronegativity difference between the two friction layers and change the surface topography of the friction layer material.^[^ ^41^ ^]^ However, these approaches often incur higher manufacturing and time costs. Another common method involves enlarging the size of TENG devices to increase surface contact area; however, this leads to larger footprints and higher production expenses. Transforming 2D planar devices into 3D structures has proven effective in addressing this issue. By designing the friction layer for double‐sided contact, TENG devices can optimize space utilization and enhance surface contact area.^[^ ^42^, ^43^ ^]^ While traditional origami technology is designed for double‐sided contact.^[^ ^44^, ^45^, ^46^, ^47^ ^]^ Existing structures exhibit relatively low space utilization rates and lack suitable driving mechanisms, resulting in insufficient friction layer contact and limited device output.^[^ ^48^, ^49^, ^50^ ^]^ Hence, it is necessary to design a suitable device to integrate a large number of units in a limited space and drive them to work under the action of water waves to achieve higher output performance.

In this work, a swinging origami structure of the TENG (SO‐TENG) was designed and fabricated to collect water wave energy. By using origami as the monomer structure of SO‐TENG, the surface contact area of TENG can be effectively improved in a limited space. At the bottom of the oscillating body, a swing structure for placing heavy objects is designed as a driving device, which can effectively collect water wave energy. Firstly, the thickness of the friction layer material and the matrix material was optimized. Next, the effects of different origami quantities, lead block weights, and various movements on the output performance were systematically measured. Finally, SO‐TENG was applied to power small electronic devices in a water‐wave environment and provided effective corrosion protection for Q235 carbon steel, demonstrating the wide range of applications of TENG in marine environments.

## 2 Results and Discussion

### 2.1 Structure and Working Principle of the SO‐TENG

**Figure 1:**
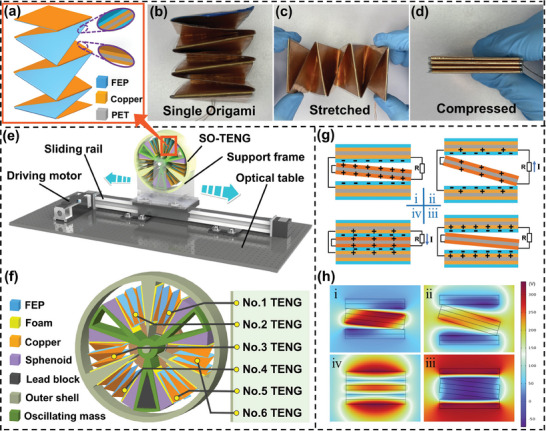
The 3D drawing, production process, and shape of origami monomer. a) A 3D diagram of an origami monomer illustrated with an enlarged cross‐section of a sandwich structure. b) Optical photo of origami monomer in its natural state. c) Optical photo of an origami unit in a stretched state. d) Optical photo of an origami unit under compression. Working process, structure, and working principle of SO‐TENG. e) Schematic diagram of SO‐TENG driven by a servo motor. f) Schematic diagram of the structure of SO‐TENG. g) Diagram of the working principle of the No. 1 origami TENG. h) The COMSOL software was used to simulate and evaluate the different working processes of the No. 1 origami TENG.

The origami structure has good elasticity and a compact design, with a large contact area between friction layers. **Figure** **1**a shows a 3D view of a single origami TENG (O‐TENG). The detailed manufacturing process is shown in Figure S1 (Supporting Information), in which two polymer strips are folded repeatedly to make O‐TENG. Both polymer strips are composed of copper /PET/ copper sandwich structures, as shown in the inset. One polymer strip serves as the friction layer, while the other acts as the electrode. A layer of friction material is applied to the copper electrode, as depicted in the inset. The optical photos of the folded origami are shown in Figure 1b. Figure 1c,d displays optical photos of the O‐TENG during stretching and compression, which exhibits excellent elastic performance. The stretching or compression process only requires a small driving force, making it easy to harvest energy from different environmental forms.

Figure 1e illustrates a schematic diagram of the measurement system for the designed swinging origami TENG device used to test its electrical output performance. The enlarged view of the SO‐TENG in the system is shown in Figure 1f. The SO‐TENG consists of a stator housing with three baffles and a three‐blade oscillating body, which is placed between each baffle and the oscillating body blades. The design incorporates a sphenoid feature to ensure full contact inside each origami, thereby enhancing the surface contact area between the friction layers. Additionally, a lead block is positioned within the hollow section of the bottom fan blade, enabling the swinging body to oscillate under external excitation, utilizing the inertia of the lead block to make the TENG work.

Upon external triggering, the prepared SO‐TENG oscillating body undergoes swinging motion due to inertia. This reciprocating oscillation causes the origami paper to experience stretching and compression movements. Illustrated in Figure 1g‐i–iv, the working mechanism of the No. 1 origami TENG begins with an initial equilibrium of charges (stage i). Upon stretching (stage ii), electrons flow from the copper electrode to the copper friction layer via the outer circuit. Subsequently, during the rebound motion, equilibrium is reached (stage iii). Driven by inertia, the oscillating body continues its motion, compressing the No. 1 origami TENG (stage iv), facilitating electron flow from the copper friction layer back to the copper electrode. This completes a cycle of work as the device returns to its initial position.

For a more in‐depth analysis of its working mechanism, the COMSOL finite element analysis software was used to simulate the operation of the No. 1 origami TENG in one cycle, as shown in Figure 1h. In the initial state, the two friction layers are separated, establishing an electrostatic equilibrium. As the origami is stretched, an induced electromotive force is generated, disrupting the initial electrostatic equilibrium. When the origami is stretched to its maximum, it reaches a new electrostatic equilibrium state. Subsequently, a rebound motion occurs as the origami is compressed. During the compression process, an induced electromotive force is generated, and when fully compressed, it reaches a new electrostatic equilibrium state.

### 2.2 Material Optimization of the SO‐TENG and Its Performance

In the process of stretching and compression, the thickness of the friction layer material and the substrate material of the origami paper have a significant impact on its output. Different friction layer materials exhibit substantial differences in electronegativity, leading to notable variations in output performance. The thickness of the PET substrate, which serves as the origami paper base, can affect the flexibility and elasticity of the origami paper. the swinging motion of the device influences the stretching and compression of the origami paper, and it also affects the folding degree and contact level of the paper. Consequently, this influences the surface contact area between the friction layers, thereby affecting the electrical output performance of the device. In the subsequent experiments, 12 origami papers with alternating contact and separation were placed on the No.1 and No.2 TENGs, as shown in Figure S2 (Supporting Information).

**Figure 2:**
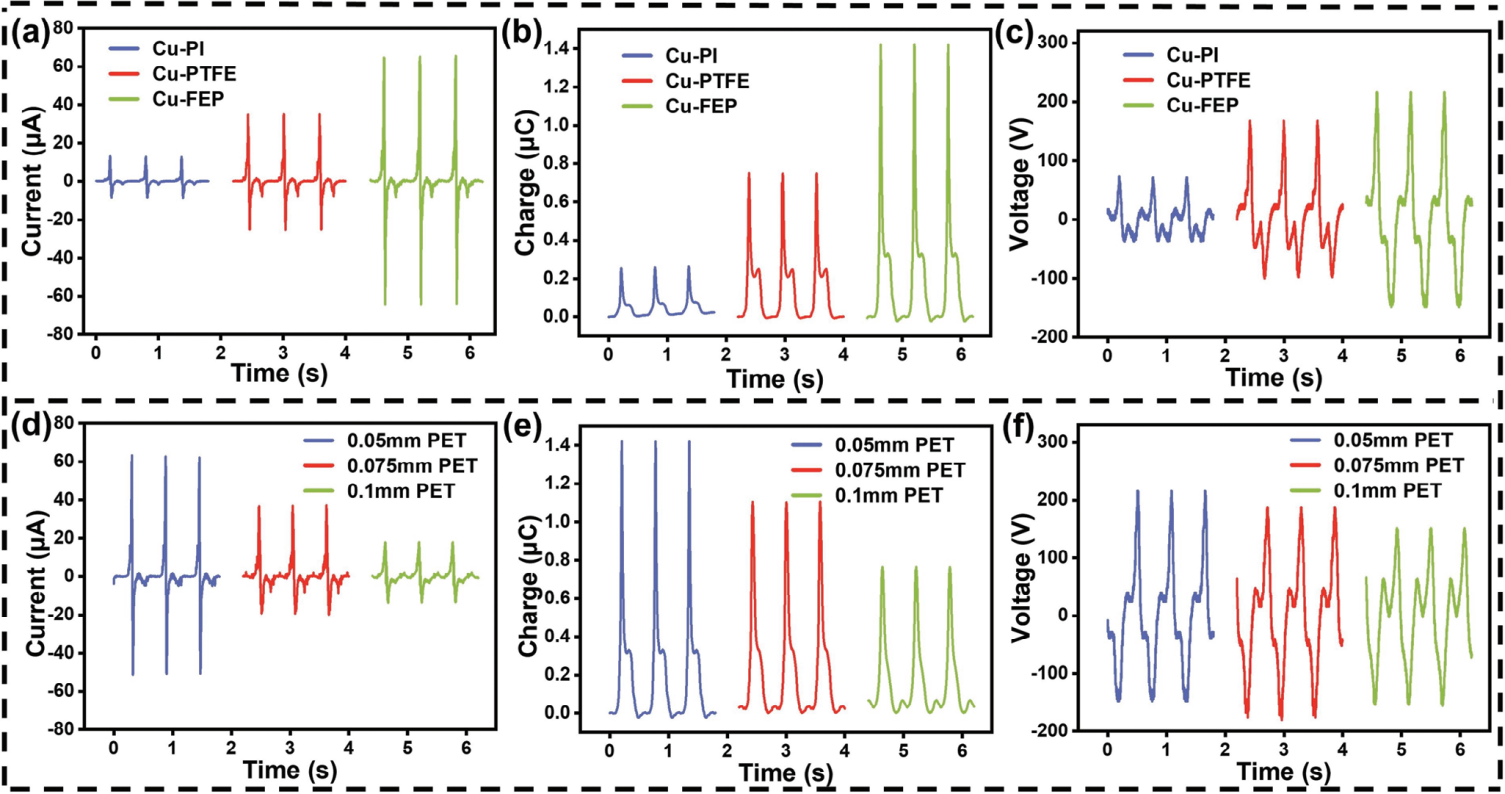
Optimization of friction materials for SO‐TENG. Output performance of the No.1 TENG under different friction layer materials in SO‐TENG: a) Short‐circuit current, b) Transferred charge. c) Open‐circuit voltage. Output performance of the No.1 TENG under different thicknesses of PET in SO‐TENG: d) Short‐circuit current, e) Transferred charge. f) Open‐circuit voltage.

Several common friction layer materials were selected: polyimide (PI), polytetrafluoroethylene (PTFE), and fluorinated ethylene propylene (FEP), and the short‐circuit current, transferred charge and open‐circuit voltage of the No.1 TENG were tested. The results are shown in **Figure** **2**a–c. It can be seen that FEP and copper (Cu) are used as friction layer materials to produce a maximum output of 65 µA, 1.5 µC, and 220 V. This is attributed to the larger electronegativity difference between these two friction layer materials. In the subsequent experiments, FEP and Cu were chosen as friction layer materials for further study.

Several thicknesses of PET substrate were selected, and the number of folded sides of origami was the same for different thicknesses, and the output performance of the SO‐TENG was tested. The results are shown in Figure 2d–f. It can be observed that the output is highest when the PET substrate material has a thickness of 0.05 mm. During the folding process of the origami paper, the 0.05 mm thick PET substrate exhibits better folding and contact effects, resulting in a larger surface contact area between the friction layer materials. Consequently, 0.05 mm thick PET was chosen as the base material for origami paper in subsequent experiments.

Based on the results of the above experiments, we optimized the friction layer material and PET matrix thickness of origami paper to enhance the device's electrical output performance. FEP and Cu were selected as the friction layer materials, and 0.05‐mm‐thick PET was selected as the base material of origami paper.

### 2.3 Structure Optimization of the SO‐TENG and Its Performance

**Figure 3:**
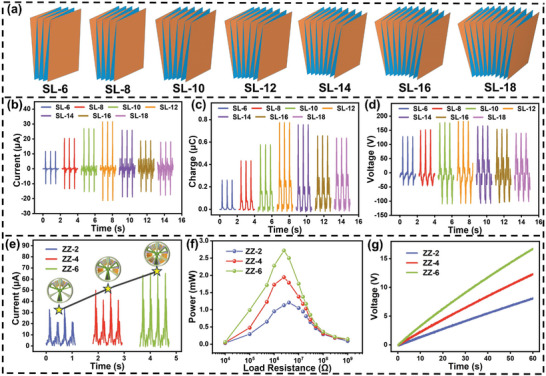
Optimization of SO‐TENG. a) 3D diagram of origami paper for SO‐TENG with different contact‐separation quantities. Output performance of No.1 TENG under different contact–separation quantity of SO‐TENG origami paper: b) short‐circuit current, c) transfer charge, d) open‐circuit voltage. Output performance of SO‐TENG after rectification under three different origami quantities: e) output current, f) the output power under different external loads. g) Charging a 47 µF capacitor.

To fully utilize the space and enhance the output performance of the SO‐TENG, the number of contact‐separations of origami paper is optimized. As shown in **Figure** **3**a, the 3D diagram of different contact‐separation numbers of origami paper and the corresponding optical photos are shown in Figure S3a (Supporting Information). The number of origami contact‐separations is 6, 8, 10, 12, 14, 16, 18. Due to the different contact separation quantities, the thickness of the origami is inconsistent in the compressed state. Therefore, different sphenoid is designed for origami with different amounts of contact‐separation. To ensure that the working process of the SO‐TENG device can be fully compressed, the specific working process, such as the Video S1, is in the Supporting Information. Different sphenoid sizes are shown in the photos in Figure S3b (Supporting Information). Subsequently, six origami papers were created for each contact‐separation number and positioned accordingly (1‐6). The results, depicted in Figure 3b–d, illustrate a trend where the output performance of the No. 1 origami paper initially increases with the contact‐separation number and then decreases. Specifically, among the 12 contact‐separation quantities tested, the maximum output reaches 33 µA, 0.75 µC, and 185 V. This phenomenon arises due to the intricate interplay between the contact area, number of contacts, and contact stress within the TENG system operating in contact separation mode. The origami paper with different amounts of contact separation is placed in the same space, and the contact stress decreases with the increase of the origami contact area. This relationship diagram between contact area and contact stress of the No. 1 origami TENG in different contact separation quantities was tested, as shown in Figure S4 (Supporting Information). Notably, when the number of contact separations of origami increases from 6 to 12, the impact of origami contact area on the device's electrical output performance surpasses that of contact stress. Conversely, when the number of contact separations of origami increases from 12 to 18, the influence of origami contact stress is greater than the effect of contact area on the electrical output performance of the device. Therefore, it is concluded that with the increase in the number of origami monomer contact separation, the electrical output performance of the device first increases and then decreases. When the number of origami contact separations is 12, the electrical output performance of the device is the highest. Therefore, in the experiments aimed at enhancing the device's electrical output performance, origami paper with 12 contact‐separation number was selected for further investigation.

Subsequently, the effect of origami quantity on the output performance of TENG devices was studied. Figure S5a (Supporting Information) shows the 3D representation of three different quantities of origami, represented as ZZ‐2, ZZ‐4, and ZZ‐6. Due to the elasticity of origami paper, with the increase of the number of origami papers, the strength between origami papers weakens and the corresponding force decreases, so the output performance of a single origami TENG weakens. This is because the electrical output performance of the contact separation mode TENG is greatly affected by the force. The electrical output performance of different amounts of origami paper after rectification is tested. Figures 3e and S5b (Supporting Information) respectively show the output current and output voltage of the TENG device after rectification. It can be seen that with the increase of the amount of origami, the output current of the TENG device increases after rectification, while the output voltage remains relatively constant. This is because the circuit after rectification is in a parallel state, the current is superimposed, and the voltage is not superimposed. In addition, on the other hand, according to TENG's theoretical basis, charge transfer mainly depends on the surface contact area and surface charge density between the friction layers. Under the same excitation conditions, increasing the amount of origami will increase the surface contact area between the friction layers. Current is defined as the amount of charge transferred per unit time. The following formula is shown:

(1) $$I=\frac{dQ}{dt}$$

Therefore, as the number of origami papers increases, the surface contact area also increases, leading to an increase in friction charge generation during the contact process. Consequently, the output current increases. However, the current output does not increase proportionally because the slight increase in resistance due to the higher number of origami papers has a relatively small impact. The output voltage remains essentially unchanged, and the voltage has no relationship with the charge transfer density. Therefore, with an increase in the number of origami papers, the output voltage remains relatively constant. The resistive load behavior of origami papers in different quantities was studied, and Figure 3f illustrates the variation in the rectified output power of the TENG with different numbers of origami papers as the external load changes. The power calculation for the TENG is given by the following formula,

(2) P=UI

When the number of origami papers increases from 2 to 6, the output power increases from 1.16 to 2.76 mW. Furthermore, the increased number of origami papers also enhances the charging speed of the capacitor, as depicted in Figure 3g. ZZ‐6 exhibits the fastest charging speed, being able to charge a 47 µF capacitor to 16.8 V within 60 s, nearly 2.5 times faster than ZZ‐2. It has been proved that the application of an origami structure in the oscillating device can effectively collect energy, and multiple origami structures can be arrayed in the oscillating device to improve its electrical output performance.

**Figure 4:**
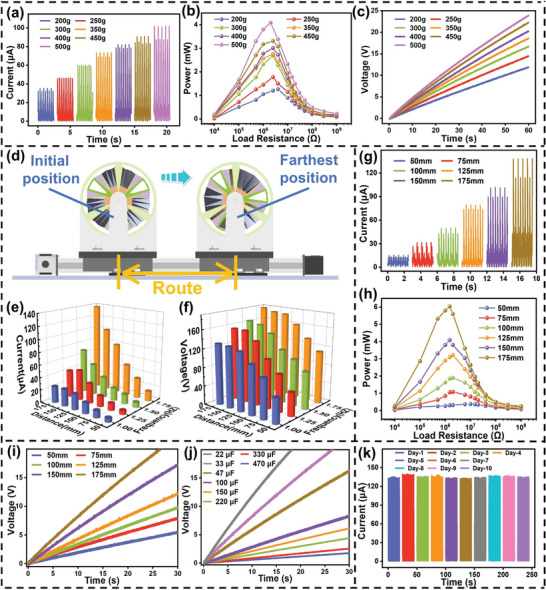
Optimization of SO‐TENG and testing of electrical output performance. a) Rectified output current of SO‐TENG under different weights of lead blocks. b) Output power under different external loads after rectification under different weights of lead blocks driving SO‐TENG. c) Charging a 47 µF capacitor after rectification under the drive of weights of different lead blocks. Simulate the output performance of SO‐TENG water wave energy harvesting in an ocean environment. d) 3D schematic of the testing principle for energy harvesting by SO‐TENG. The output performance of SO‐TENG under different reciprocating stroke and frequency: e) output current, f) output voltage. The output performance of the SO‐TENG under different reciprocating strokes after rectification at a reciprocating frequency of 1.75 Hz: g) output current, h) external load output power, i) charging a 33 µF capacitor, j) charging capacitors of different capacitances. k) durability and stability testing of SO‐TENG device over ten continuous days.

To enhance the application of the TENG device for blue energy collection in marine environment and optimize the electrical output performance of the SO‐TENG, further optimizations were pursued. Lead weight plays a very important role in the energy harvesting process of TENG devices with swinging origami structure. It is used to collect energy and then drive the origami structure to complete the contact separation movement to achieve electrical energy output. In theory, the greater the weight, the greater the inertia during motion. Figure S6 (Supporting Information) shows a 3D view of SO‐TENG with optical images of lead blocks of different weights. The seven lead blocks with different weights have the same cross‐sectional area, and as the weight increases, the length of the lead block increases proportionally. In order to improve the electrical output performance of the device, under a reciprocating frequency of 1.75 Hz and a stroke of 150 mm, we tested the electrical output performance of several different weight lead blocks. **Figures** **4**a and S7 (Supporting Information) show the rectified output current and output voltage, respectively; it can be observed that the output current of SO‐TENG increases with the weight of the lead block. When the weight of the lead block is 500 g, the current can reach 102 µA. This phenomenon can be attributed to the heightened inertia with increased weight, resulting in a more compact surface contact area between the friction layers. Consequently, the friction charge generated during the contact process amplifies, leading to a corresponding increase in output current. The output voltage also increases with the weight of the lead block, but the increase is relatively slow. Additionally, the resistive load behavior under different weights of the lead block is studied, as shown in Figure 4b. Its performance is positively correlated with the weight of the lead block. When the weight of the lead block is 500 g, with a matching resistance of 2 MΩ, the maximum peak power is 4.085 mW. The impact of lead block weight on the charging performance of SO‐TENG after rectification was then compared, as shown in Figure 4c. As the weight of the lead block increases, the charging speed increases. A 500‐g lead block has the fastest charging speed, being able to charge a 47 µF capacitor to 24 V within 60 s, which is twice as fast as the 200‐g lead block. Consequently, for further experimentation aimed at optimizing the device structure and enhancing its electrical output performance, a 500 g lead block was chosen.

In order to evaluate the output performance of SO‐TENG, the influence of different reciprocating strokes and frequencies on the output performance of SO‐TENG was tested. Figure 4d shows a schematic diagram of its working process, and SO‐TENG is driven by a servo motor. As shown in Figure 4e,f, we tested the dependence of the output current and output voltage of SO‐TENG on different reciprocating strokes and frequencies. It can be observed that with the increase of reciprocating stroke and frequency, the output performance of SO‐TENG improves. This indicates that with the increase of reciprocating stroke and frequency, the folding degree of the paper becomes more complete, increasing the surface contact area between the friction layers, resulting in better output performance. Subsequently, the output performance of SO‐TENG after rectification was specifically tested under different reciprocating strokes at a frequency of 1.75 Hz, as shown in Figures 4g and S8 (Supporting Information). With the increase of reciprocating stroke, the output current and output voltage increase. At a stroke of 175 mm, the output can reach 138 µA and 188 V. This is because with the increase of reciprocating stroke, the relative motion speed increases, resulting in better folding degree and contact effect of the paper. At a matching internal resistance of 15 MΩ, the maximum peak power can reach 6.035 mW, and the peak power density can reach 2.62 W m^−2^, demonstrating extremely high output performance (Figure 4h). Subsequently, the charging performance of the rectified SO‐TENG under different reciprocating strokes is compared, as shown in Figure 4i. As the reciprocating stroke increases, the faster the capacitor is charged. A 33 µF capacitor can be charged to 20 V in 27 s at a 175‐mm stroke. In addition, different capacitors are charged, as shown in Figure 4j. As the capacitance increases, the capacitor charging speed decreases, demonstrating good charging performance for different capacitors. The stability of TENG devices is crucial. To evaluate the durability and stability of the SO‐TENG device, the current output after 1 h of rectification was continuously tested for 10 d. As shown in Figure 4k, the continuous output current for 10 d remained around 138 µA, which has good output stability. The unique advantages of applying origami structures to oscillating devices are demonstrated.

### 2.4 Application of SO‐TENG in a Water Wave Environment

**Figure 5:**
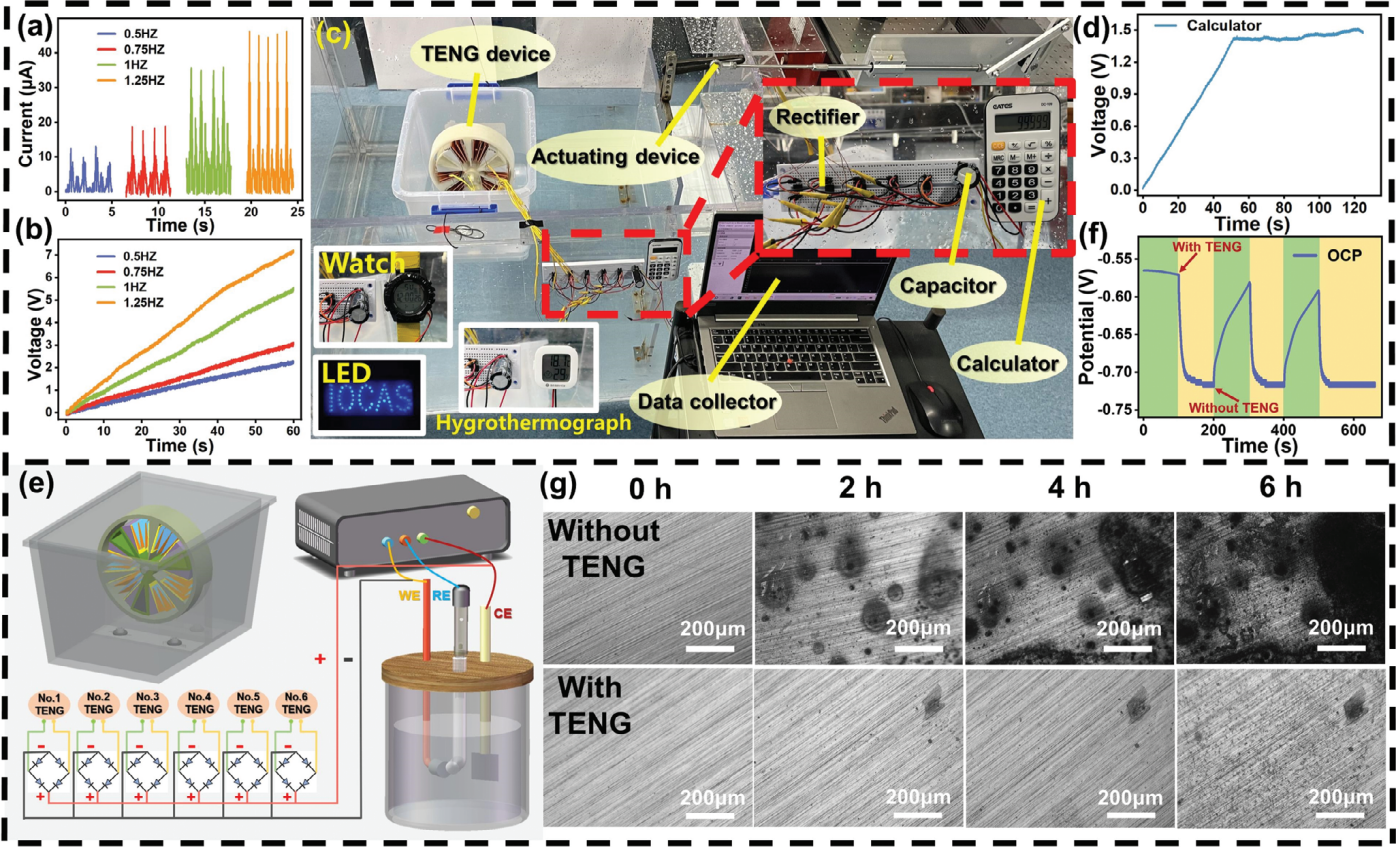
Application of SO‐TENG wave energy harvesting in the simulated real ocean environment. a) Output current after rectification of SO‐TENG at different water wave frequencies. b) Charging of a 47 µF capacitor after rectification of SO‐TENG at different water wave frequencies. c) Real testing environment for SO‐TENG wave energy harvesting. d) Charging diagram for powering the calculator. e) Schematic diagram of the cathodic protection system for SO‐TENG. f) Curve showing the variation of open‐circuit potential. g) Metallographic images of Q235 carbon steel after 6 h with and without DR‐TENG protection.

To simulate and test the performance of the SO‐TENG device in collecting wave energy in a real ocean environment, we placed a plastic box containing the SO‐TENG device into a water tank environment in the laboratory to simulate wave motion. The water waves were generated by a motor driving a wave‐making board back and forth. **Figure** **5**a shows the output current after rectification of the SO‐TENG device is shown for four different water wave frequencies. It can be observed that the current increases with the increase in water wave frequency. This is because as the water wave frequency increases, the contact between the friction layers of individual TENGs in the motion process of the SO‐TENG becomes more sufficient, leading to higher current output. Figure 5b illustrates the charging of a 47 µF capacitor after rectification of the SO‐TENG device under four different water wave frequencies. At a water wave frequency of 1.25 Hz, the SO‐TENG device can charge a 47 µF capacitor to 7 V within 60 s. It is further demonstrated that in a certain frequency range of water wave, the output performance of SO‐TENG is better with the increase of water wave frequency. By charging the capacitor, the stored energy can drive small electronic devices. Figure 5c shows the test diagram of SO‐TENG under a real water wave environment. Figure 5d shows the voltage variation diagram of charging a 150 µF capacitor driven by a water wave frequency of 1 Hz. It can be seen that SO‐TENG charged the 150 µF capacitor to 1.4 V within 50 s, making the calculator work normally. The voltage remains stable with continuous operation of the SO‐TENG, enabling the calculator to function normally as illustrated in Video S2 (Supporting Information). In addition, under the same water wave conditions, the SO‐TENG can be driven to power small watch, hygrothermograph, and LEDs, as shown in the illustration in Figure 5c. The circuit diagram for charging electronic devices with the SO‐TENG device is illustrated in Figure S9 (Supporting Information).

As an economical and environmentally friendly energy source, TENG can harvest wave energy in marine environments and convert it into electrical energy. The application of the generated electrical energy in the corrosion protection of metals shows promising prospects, and many researchers have been actively involved in this field.^[^ ^51^, ^52^, ^53^, ^54^, ^55^, ^56^ ^]^ The schematic diagram of the cathodic protection system for SO‐TENG is shown in Figure 5e. The alternating current output during the operation of SO‐TENG is converted into direct current output through the rectification bridge. During the operation of SO‐TENG, electrons transfer to the surface of Q235 carbon steel to effectively protect it.

In electrochemical experiments, open circuit potential (OCP) is an important parameter used to evaluate the effect of metal cathodic protection.^[^ ^57^, ^58^, ^59^, ^60^, ^61^ ^]^ Subsequently, the TENG equipment was driven to protect the Q235 carbon steel in a real water wave environment, and its OCP changes were tested, as shown in Figure 5f. Initially, when the TENG is not connected, the OCP value of the Q235 carbon steel is ‐565 mV. Upon connecting the TENG at 100 s, the OCP value of the Q235 carbon steel rapidly drops to ‐720 mV, indicating that the Q235 carbon steel provides effective protection when connected to the TENG. Here, the OCP drops by 155 mV, and after disconnecting the circuit at 200 s, the OCP value recovers. In general, achieving a lower OCP value is imperative for optimal cathodic protection. Hence, the application of SO‐TENG demonstrates a commendable protective effect on Q235 carbon steel in a real water wave environment. To further illustrate the corrosion protection provided by the SO‐TENG on Q235 carbon steel, metallographic microscope images provide a more intuitive view of the corrosion situation of Q235, as shown in Figure 5g. It is evident that the extent of corrosion for Q235 carbon steel without SO‐TENG protection intensifies over time, while the Q235 carbon steel protected by the SO‐TENG shows only slight corrosion traces after 6 h of immersion. These findings demonstrate the promising applicability of SO‐TENG for wave energy harvesting in marine environments.

## 3 Conclusion

In summary, we have designed and manufactured a TENG with a swinging origami structure for the collection of wave energy. Utilizing origami as the monomer structure of the device, the device can effectively improve the surface contact area of the TENG in a limited space and greatly improve the output performance of the TENG device. The lead block is placed on the bottom fan blade of the swing body of the swing device so that the swing body can make the reciprocating swing motion due to the influence of inertia under the trigger of the water wave so that the contact separation motion between the friction layers of the origami structure can occur. It can greatly improve the folding degree and contact degree of origami, with an output of up to 138 µA and a peak power density of 2.62 W m^−2^ under a weight of 500 g, with good output performance. When operating under low‐frequency water waves, the device exhibits reliable power generation capabilities and can power small electronic devices in underwater environments. When applied to corrosion protection of metals, the open‐circuit potential of Q235 carbon steel, protected by TENG, is reduced by approximately 155 mV, showing effective corrosion protection. This indicates a broad application prospect for TENG in large‐scale wave energy harvesting and the realization of self‐powered ocean sensors in marine environments.

## 4 Experimental Section

### Fabrication of a Single Origami TENG

The detailed manufacturing process of a single origami TENG (O‐TENG) is depicted in Figure S1 (Supporting Information). Two polymer strips are repeatedly folded to create the O‐TENG. In this process, polyethylene terephthalate (PET) material, known for its good flexibility, is used as the substrate material between the two polymer strips. Both polymer strips consist of a layered composite structure with widths of 48 mm/50 mm/48 mm for copper/PET/copper, respectively. One polymer strip serves as the friction layer, while the other serves as the electrode. A friction layer material covering a width of 50 mm is applied to the copper electrode. The friction layer materials include polytetrafluoroethylene (PTFE), fluorinated ethylene propylene (FEP), and polyimide (PI). Figure 1c,d illustrates the stretching and compressing states of the fabricated O‐TENG device.

### Fabrication of Swing Origami‐Structure Based TENG

The fabrication of the swing origami TENG (SO‐TENG) is primarily composed of an outer shell, oscillating body, sphenoid, lead block, bearings, and six O‐TENGs. The outer shell and oscillating body of the SO‐TENG were manufactured using ultraviolet‐cured resin through 3D printing technology. The connection between the outer shell and the oscillating body is achieved using a shaft and bearings. The outer shell (outer diameter, 186 mm; height, 50 mm) contains three baffles to control the range of motion of the oscillating body, and a slot is designed on the back of the outer shell to accommodate the bearings. The oscillating body consists of three hollow blades, and the sphenoid is manufactured using 3D printing fused deposition modeling technology positioned at the ends of the oscillating body blades. The size of the sphenoid is designed according to the compressed state of origami, and the size is different for different folds of origami.

Six O‐TENGs are placed between the outer shell and the oscillating body, attached at both ends with double‐sided foam tape, forming six triboelectric nanogenerator units. The lead block is placed in the hollow part of the bottom fan blade. The material of the lead block is lead with higher density. Its cross‐section shape is an isosceles trapezoid, and the thickness is different according to the different weight. The cross‐section area is 4.5 mm at the top, 24.5 mm at the lower bottom, and 39 mm in height. Depending on the inertia of the lead block, the oscillating body moves under external excitation. A stand is designed with a semi‐circular bottom (diameter, 186 mm). The prepared TENG device is placed and then fixed on the servo motor to simulate the output performance of the TENG device in the marine environment.

In the application part, a saturated NaCl solution with a mass fraction of 3.5 wt% was prepared to simulate the marine environment. A three‐electrode system was chosen for the experiment, with a mercury electrode as the reference electrode, a platinum electrode as the opposite electrode to connect the positive pole of the rectifier bridge, and a 5 mm × 5 mm Q235 carbon steel used as the working electrode to connect the negative pole of the rectifier bridge.

### Characterization and Measurements

The TENG operation is driven by a servo motor (60A6N‐M01330, HAIJIE JIACHUANG, China). Electrical output measurement of TENG devices: oscilloscope (DS12022‐E, RIGOL, China) was employed to measure the voltage of the TENG; The electrometer K6514 (Keithley6514, Tectronix, USA) was employed to measure the current and transfer charge of TENG. The TENG in metal corrosion protection applications was assessed in a three‐electrode system utilizing the electrochemical workstation (CHI760E, Shanghai Chenhua, China). A metallographic microscope (Axiocam 105 color, ZEISS, Germany) is employed to observe the corrosion morphology of Q235 carbon steel.

## Conflict of Interest

The authors declare no conflict of Author.

## Supporting information

Supporting Information

Supplemental Video 1

Supplemental Video 2

## Data Availability

The data that support the findings of this study are available from the corresponding author upon reasonable request.
