# Supplementary material for: Swing Origami‐Structure‐Based Triboelectric Nanogenerator for Harvesting Blue Energy toward Marine Environmental Applications
Source: Adv Sci (Weinh). 2024 Apr 11;11(23):2401578. doi: 10.1002/advs.202401578 (PMC11187886; doi:10.1002/advs.202401578)
Supplement: Supplementary file 1 — Supporting Information [file ADVS-11-2401578-s001.pdf]

ADVANCED SCIENCE
Open Access

Supporting Information

for *Adv. Sci.*, DOI 10.1002/advs.202401578

Swing Origami-Structure-Based Triboelectric Nanogenerator for Harvesting Blue Energy toward Marine Environmental Applications

Weilong Liu, Xiutong Wang, Lihui Yang, Youqiang Wang, Hui Xu, Yanan Sun, Youbo Nan, Congtao Sun, Hui Zhou and Yanliang Huang*

Supporting Information

Swing Origami-Structure-Based Triboelectric Nanogenerator for Harvesting Blue Energy toward Marine Environmental Applications

Weilong Liu[1,2,3], Xiutong Wang[1,4,*], Lihui Yang[1], Youqiang Wang[3], Hui Xu[1], Yanan Sun[1], Youbo Nan[1,2,4], Congtao Sun[1], Hui Zhou[1], Yanliang Huang[1]

[1] Key Laboratory of Advanced Marine Materials, Institute of Oceanology, Chinese Academy of Sciences, Qingdao, 266071, China

[2] Institute of Marine Corrosion Protection, Guangxi Key Laboratory of Marine Environmental Science, Guangxi Academy of Sciences, Nanning, 530007, China

[3] School of Mechanical and Automotive Engineering, Qingdao University of Technology, Qingdao, 266525, China

[4] University of Chinese Academy of Sciences, Beijing, 100049, China

* Corresponding author.

E-mail address: wangxiutong@qdio.ac.cn (X. Wang)

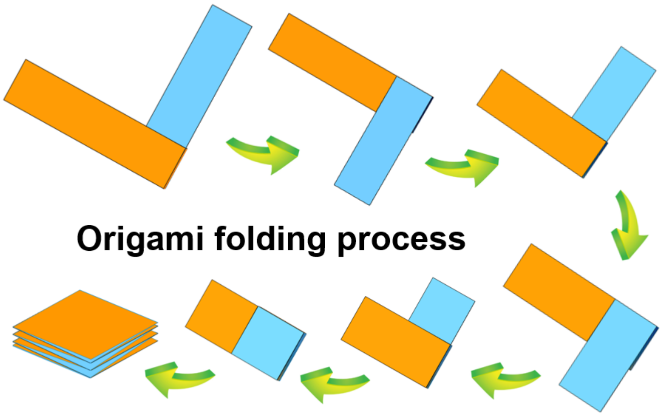

Figure S1. Folding process of origami monomer.

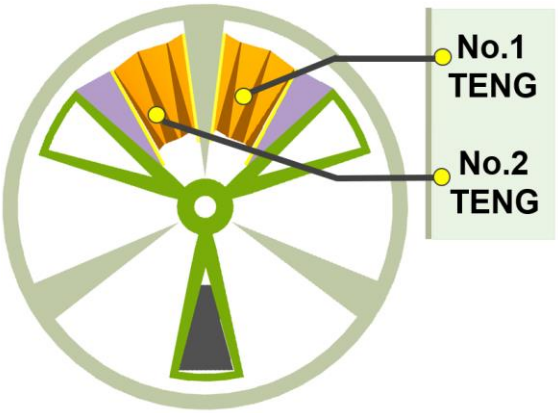

Figure S2. Schematic diagram of the SO-TENG testing setup.

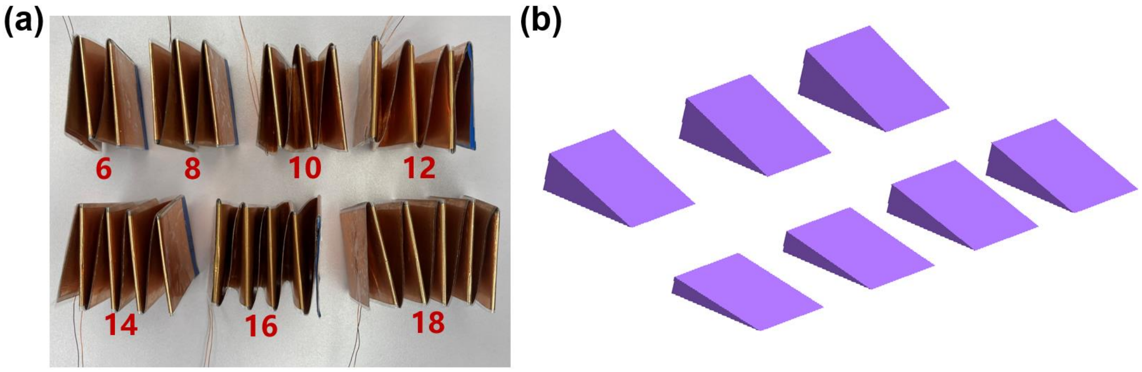

Figure S3. a) Optical photos of origami paper with different contact separation numbers for SO-TENG. b) Size photos of different sphenoid of SO-TENG.

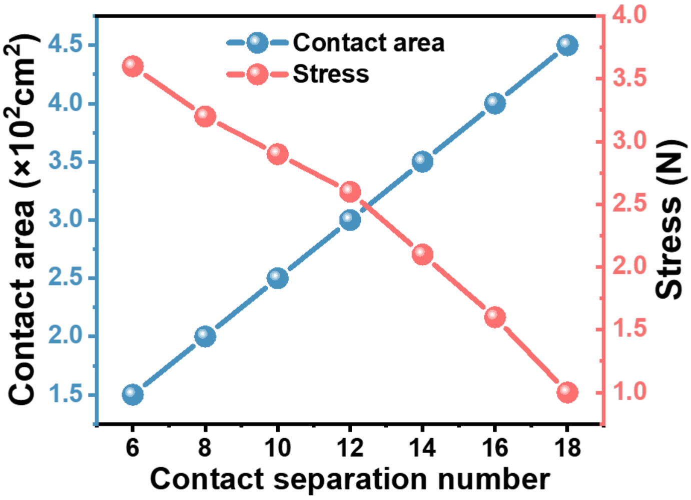

Figure S4. The relation between contact area and contact stress of origami paper with different contact separation quantity.

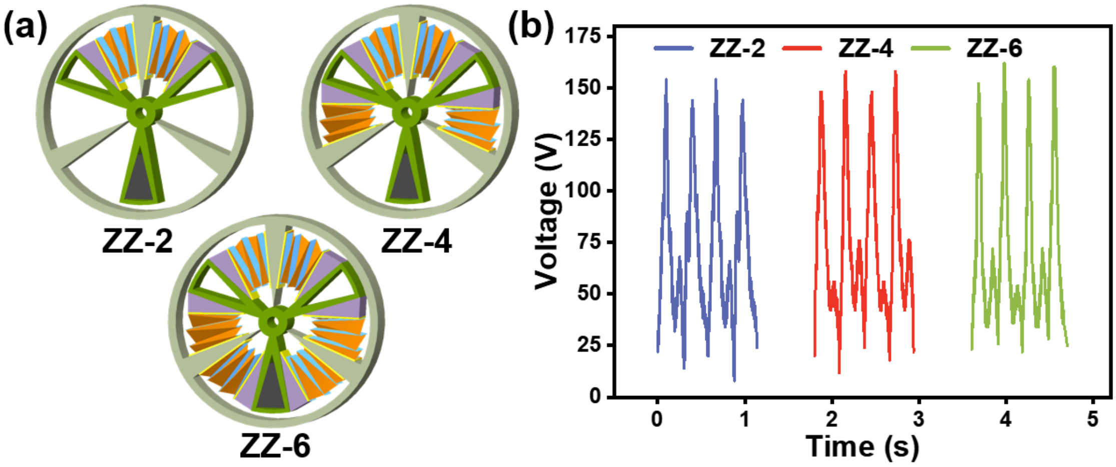

Figure S5. a) 3D schematic representation of SO-TENG with three different numbers of origami papers. b) Open-circuit voltage after finishing under three different origami quantities of SO-TENG.

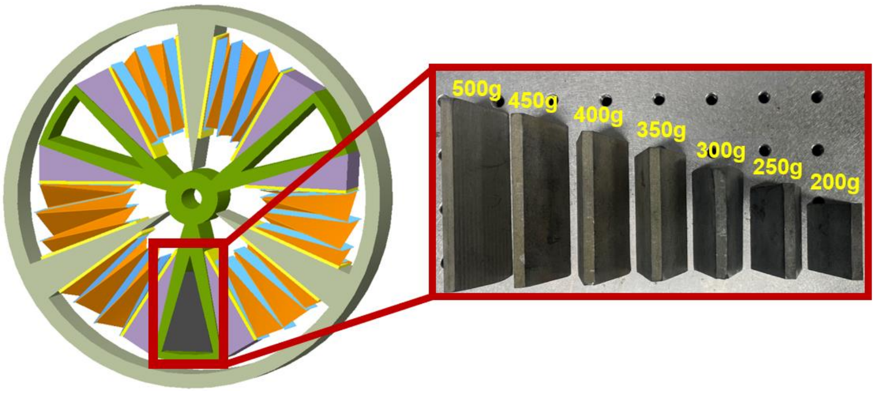

Figure S6. 3D view of SO-TENG with optical photographs of 7 lead blocks of different weights.

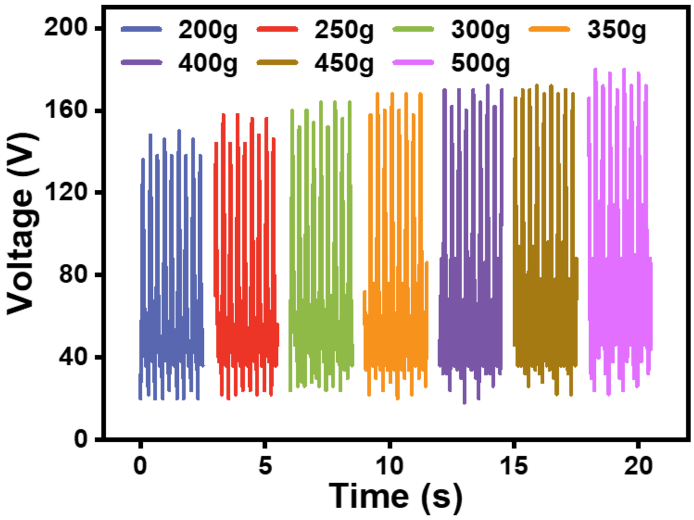

Figure S7. Rectified output voltage of SO-TENG under different weights of lead blocks.

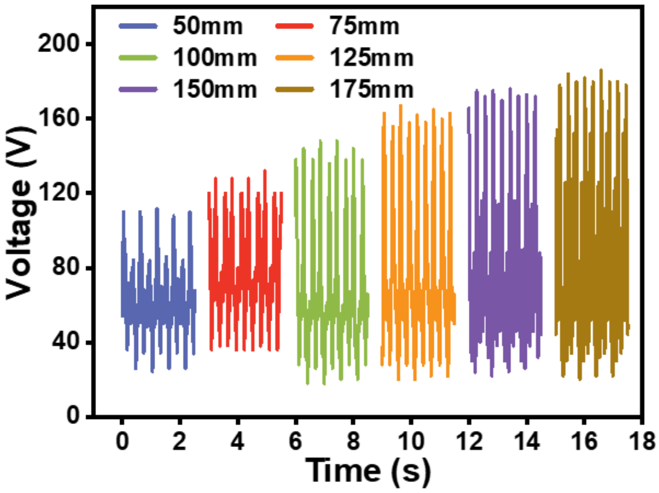

Figure S8. At the reciprocating frequency of 1.75 Hz, the output voltage of the SO-TENG with different reciprocating strokes after rectification is obtained.

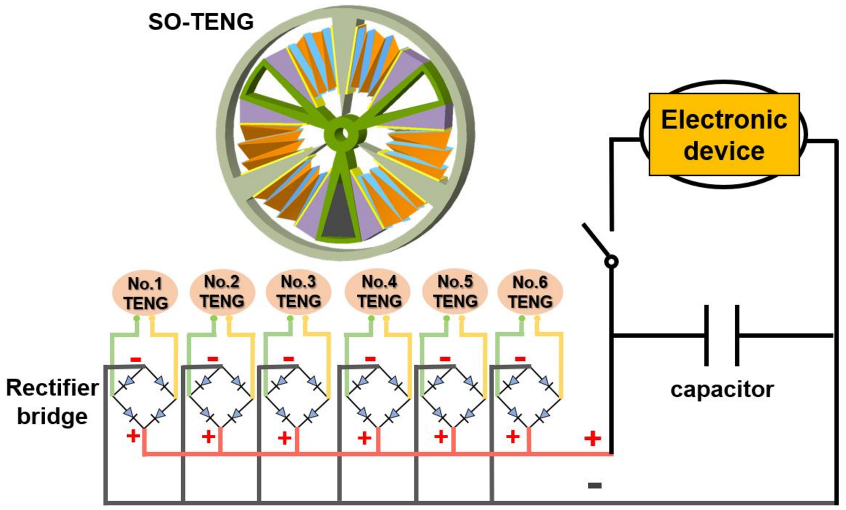

Figure S9. Circuit diagram of charging electronic device for SO-TENG device.
